# Comparing the two Greek archipelagos plant species diversity and endemism patterns highlight the importance of isolation and precipitation as biodiversity drivers

**DOI:** 10.1186/2241-5793-21-16

**Published:** 2014-09-19

**Authors:** Eleni Iliadou, Athanasios S Kallimanis, Panayotis Dimopoulos, Maria Panitsa

**Affiliations:** Department of Environmental and Natural Resources Management, University of Ioannina, Agrinio, GR-30100 Greece; Department of Environmental and Natural Resources Management, University of Patras, Agrinio, GR-30100 Greece

**Keywords:** Aegean islands, Ionian islands, Plant diversity, Plant geography, Endemic species patterns, Single island endemics

## Abstract

**Background:**

Greece has two island archipelagos, the Aegean and the Ionian, which host a rich array of plants and wildlife, particularly endemic and threatened plant species. Despite the long history of island biogeographic studies in the Aegean, similar studies in the Ionian remain limited, with the two island archipelagos rarely being compared.

**Results:**

The Aegean and Ionian archipelagos share many features, especially regarding total plant diversity, but exhibit different patterns of endemism. For instance, when considering similarly sized islands, those in the Ionian host as many as, if not more, species compared to the Aegean. In contrast, the Ionian Islands are poor in endemics (particularly narrow range endemics, such as single island or regional endemics) and threatened taxa, compared to the Aegean Islands. In the Ionian, endemics only persist on the largest islands, and form a very small proportion of the species pool, compared to the Aegean archipelago.

**Conclusions:**

The lack of endemism might be attributed to the more recent separation of the Ionian Islands from the mainland and the shorter distance separating them from the mainland. In addition, the Ionian Islands receive higher levels of precipitation and are typically covered by denser and higher vegetation than the Aegean Islands. These conditions favour greater total species richness, but tend to lead to higher numbers of common species compared to threatened and endemic taxa. This study demonstrates that both isolation and precipitation serve as biodiversity drivers, influencing plant species diversity and endemism patterns, of the two Greek archipelagos.

**Electronic supplementary material:**

The online version of this article (doi:10.1186/2241-5793-21-16) contains supplementary material, which is available to authorized users.

## Background

Islands are significant for ecological and evolutionary research, because they provide natural sites for experiments on the ecology and evolution of species [1, 2]. Islands are hotspots for species with restricted distribution (i.e. endemism hotspots), but tend to be less diverse compared to nearby mainland areas [3]. Thus, nine of the 25 global biodiversity hotspots are islands and archipelagos [4]. The Mediterranean basin represents one of these hotspots [5], with about 60% of native taxa found in this region being Mediterranean endemics [6–8].

Greece is characterized by a high degree of endemism (22.1% of its plant taxa are Greek endemics) compared to other European and Mediterranean countries of similar area [9–12]. The origin and distribution patterns of total and endemic species richness have been related to palaeogeographical patterns [13, 14]. The Greek islands are characterized by flora originating from three different continents: Europe, Asia and Africa [15]. The influence of these combined floristic elements in the local island floras seems to be more profound to the Aegean than the Ionian Islands.

Many studies have examined the factors that influence the biodiversity and endemism of the flora and fauna in the Aegean archipelago, either separately or in comparison with other archipelagos [16–22]. These studies showed that various biogeographic factors influence facets of biodiversity differently. For instance, area is the most important driver of total species richness, whereas habitat diversity and maximum elevation are more strongly associated with endemic species diversity [23–28]. Similar results have been obtained for both terrestrial vertebrate and invertebrate taxa [29–38]. However, all of these studies focused on the Aegean archipelago alone. While many studies have been conducted on the flora and fauna of the islands and islets of the Ionian archipelago, there has been limited research on the effects of biogeographic factors on plant species richness. In addition, to the best of our knowledge, there has been no comparative studies among these two Greek archipelagos.

Both archipelagos consist mainly of land bridge, continental shelf islands that, according to [3], possessed terrestrial connections to other landmasses via exposed continental shelf during periods when the sea level was low.

Both archipelagos cover a similar latitudinal range, and have a typical Mediterranean climate. However, precipitation levels are higher in the Ionian Islands (with mean precipitation 1038 mm annually and standard deviation 89) compared to the Aegean Islands (with mean precipitation 619 mm annually and standard deviation 152) according to World Clim-Global climate data (sea level values, URL: http://www.worldclim.org). The geological evolution of the Ionian Islands is comparatively simple, recently separated from the Greek mainland [39]. In contrast, the Aegean Islands are characterized by a complex geological and geographical history [40].

This study aimed to (1) examine the relationship between biogeographic factors and plant species richness in the Aegean and Ionian archipelagos, (2) estimate patterns of endemism at different scales and compare endemism rates across the two archipelagos, and (3) compare the effects of potential biogeographic factors on the insular plant species diversity of the two archipelagos, focusing on both total and endemic species richness.

## Results

The number of Aegean and Ionian Islands that host endemic and threatened taxa is shown in Table 1. Only the largest islands in both archipelagos host single island endemics (Table 1). For instance, Crete (the largest island in the Aegean) has 176 single island endemics and Euboea (the second largest island) has 35 single island endemics.

**Table 1:** **Minimum values of A, E, Dm and Dis for both Archipelagos**

	All islands		Islands with Greek endemics		Islands with Regional endemics		Islands with single-island endemics		Islands with threatened species	
	Ae	Ion	Ae	Ion	Ae	Ion	Ae	Ion	Ae	Ion
**Number of islands**	210	31	144	10	65	8	18	4	116	20
**A (ha)**	0.04	1.3	0.09	118	0.125	426	462	3000	0.10	1.3
**E (m)**	2	12	3	22	6	421	27	230	4	12
**Di (km)**	0.03	1	0.04	1	0.04	1	5.4	1	0.040	1
**Dm (km)**	0.108	0.5	0.108	0.5	0.108	0.5	0.108	0.5	0.108	0.5
**Total species richness**	1	33	4	203	16	203	275	419	5	33

Ae = Aegean, Ion = Ionian.

**A =** Island area, **E =** Maximum elevation, **Dis =** Distance from other inhabited islands for islands and islets, **Dm =** Distance from the mainland.

The occurrence of endemics is favoured by larger island area and maximum elevation. The narrower endemics appear only in the larger islands. Only the larger islands with great elevation host endemic species in the Ionian (Table 1). In contrast, endemic species are found on much smaller islets in the Aegean. The minimum island area hosting endemics in the Ionian was two or more orders of magnitude greater than the corresponding area in the Aegean (Table 1). This difference was not recorded for total species richness.

Both archipelagos (Table 2) exhibited pronounced colinearity, especially between island area and maximum elevation (R = 0.838 for the Aegean and R = 0.915 for the Ionian). Area was strongly correlated with human impact in both archipelagos (R = 0.716 for the Aegean and R = 0.837 for the Ionian). The number of substrata was strongly correlated with Area in the Ionian archipelago (R = 0.765) but significantly in the Aegean (R = 0.498). Maximum elevation was strongly correlated to the number of substrata in the Ionian (R = 0.747) and was significantly correlated to human impact in both archipelagos (R = 0.692 for the Aegean and R = 0.655 for the Ionian).

**Table 2:** **Spearman rank correlation coefficients among biogeographic factors of islands in the two archipelagos**

		Island area	Maximum elevation (E)	Distance to inhabited island (Dis)	Distance to mainland (Dm)	Latitude (Lat)	Longitude (Lon)	Index of human impact (HI)
**Maximum elevation (E)**	Ion	0.915**						
	Ae	0.838**						
**Dis**	Ion	-0.561**	-0.615**					
	Ae	0.483**	0.362**					
**Dm**	Ion	ns	ns	ns				
	Ae	ns	ns	-0.192**				
**Lat**	Ion	ns	ns	-0.454*	ns			
	Ae	ns	ns	0.156*	-0.610**			
**Lon**	Ion	-0.460**	ns	0.689**	ns	-0.462**		
	Ae	ns	ns	-0.277**	-0.216**	-0.435**		
**HI**	Ion	0.837**	0.655**	-0.518**	ns	ns	-0.682**	
	Ae	0.716**	0.692**	0.310**	-0.198**	ns	ns	
**Geo**	Ion	0.765**	0.747**	-0.677**	ns	ns	-0.640**	ns
	Ae	0.498**	0.471**	0.339**	ns	ns	-0.232**	-0.479**

Ae = Aegean, Ion = Ionian.

*p < 0.05, **p < 0.001.

Examination of total, endemic and threatened species richness in comparison to biogeographic variables in the two archipelagos by Spearman’s correlation coefficient showed that certain patterns were similar, while others differed (Table 3). For instance, in both archipelagos, total species richness was most strongly correlated to the island area (Aegean: R = 0.850, Ionian: R = 0.928), followed by the maximum elevation of the island (Aegean: R = 0.754, Ionian: R = 0.837), human impact (Aegean: R = 0.724, Ionian: R = 0.772) and the number of geological substrata (Aegean: R = 0.477, Ionian: R = 0.744).

**Table 3:** **Spearman rank correlation coefficients of all pairwise correlations between plant species diversity and biogeographical factors for the two Archipelagos**

	A	E	Dis	Dm	Lat	Lon	HI	Geo	Total
**Total species richness (S)**									
Ion	0.928***	0.837***	-0.509*	ns	ns	-0.430*	0.772***	0.744***	
Ae	0.850***	0.754***	0.422***	ns	ns	ns	0.724***	0.477***	
**Greek endemic species (GE)**									
Ion	0.865*	0.767*	ns	ns	ns	ns	0.819*	0.805*	0.902**
Ae	0.615*	0.564*	0.272*	0.181*	ns	-0.219	0.549*	0.481*	0.656*
**Regional endemics (RE)**									
Ion	0.822*	0.786*	ns	0.736*	ns	ns	ns	ns	0.761*
Ae	0.685*	0.660*	0.299*	ns	ns	ns	0.676*	0.275*	0.708*
**Single island endemics (SIE)**									
Ion	ns	ns	ns	ns	ns	ns	ns	ns	ns
Ae	0.699*	0.799*	ns	ns	ns	ns	ns	0.483	0.721***
**Threatened species (TS)**									
Ion	0.830***	0.765***	-0.590*	ns	ns	-0.492*	0.827***	0.766***	0.878***
Ae	0.763***	0.749***	0.400***	ns	ns	ns	0.741***	0.513***	0.820***
**Threatened Endemics (TES)**									
Ion	ns	ns	ns	ns	ns	ns	ns	ns	ns
Ae	0.663***	0.718***	0.253*	ns	ns	ns	0.645***	0.403***	0.687***

Ae = Aegean, Ion = Ionian.

*p < 0.05, **p < 0.001, ***p < 0.0001.

In both archipelagos, Greek endemic and threatened species richness were strongly correlated to area. However, in the Aegean, the second strongest correlation was to elevation followed by human impact, whereas human impact was the second strongest correlation in the Ionian, followed by number of geological substrata. The number of Greek endemics and the number of threatened species appear to be strongly correlated to geological diversity in both archipelagos.

In contrast, the patterns of regional, single island endemic and threatened endemic species richness differed in the Aegean and the Ionian. Regional endemic species richness in the Aegean was strongly correlated to area, followed by human impact and elevation. Furthermore, single island endemic species richness was strongly correlated to elevation, followed by area, in the same region. In contrast, regional endemic species richness in the Ionian was strongly correlated to area, followed by elevation and distance to the mainland. The predictors of threatened species in this region were area, followed by human impact, geological substrate and elevation. Among the studied factors, none was a significant predictor of single island endemic or threatened endemic species richness in the Ionian area (Table 3).

**Figure 1:**
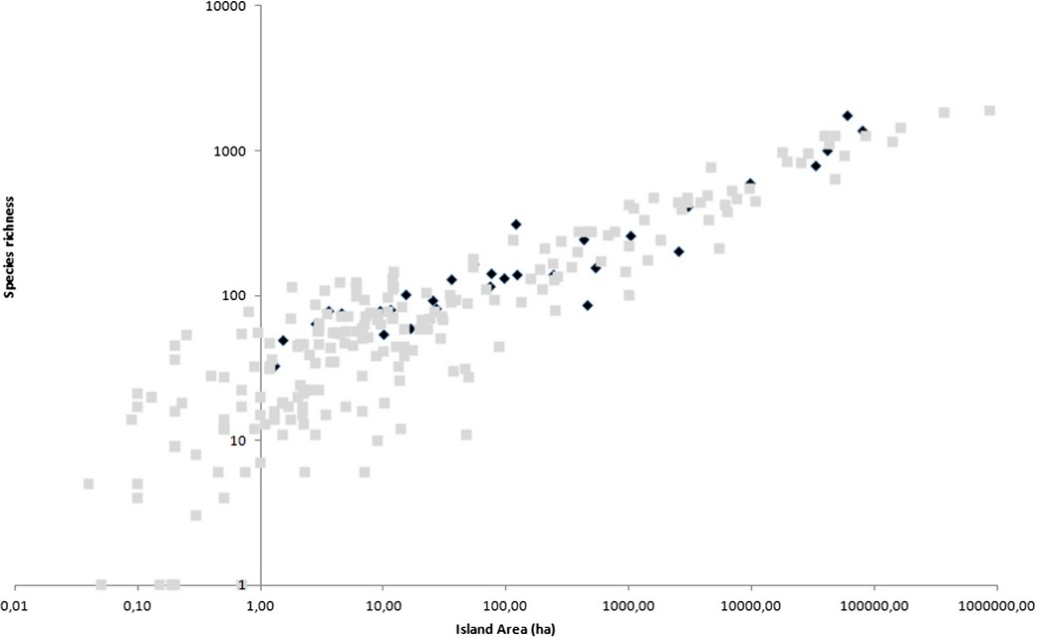
**Species-Area relationship for total species richness in the Aegean (grey squares) and Ionian (black diamonds) islands.**

**Figure 2:**
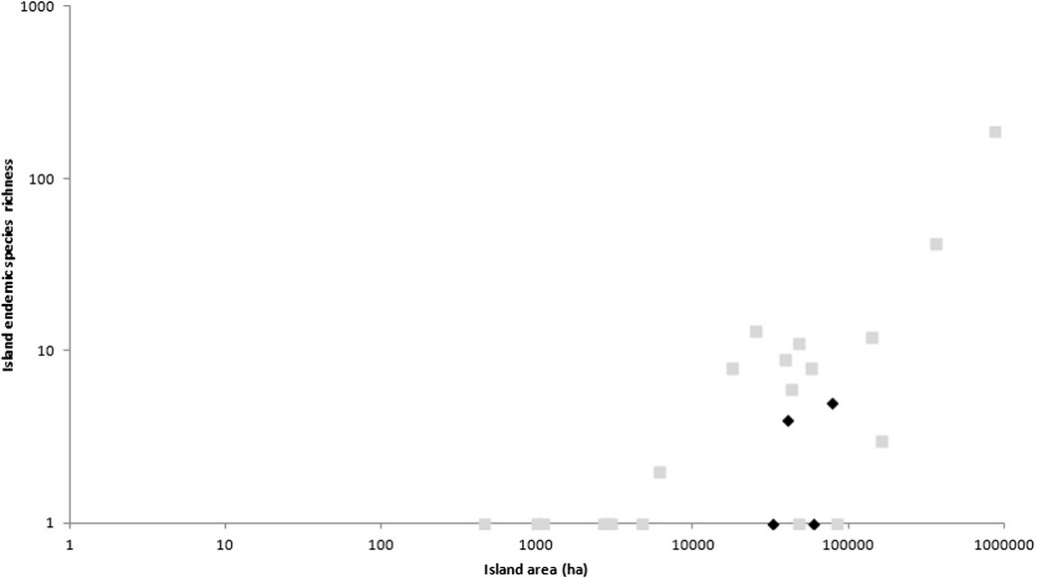
**Species-Area relationship for single island endemic species richness in the Aegean (grey squares) and Ionian (black diamonds) islands.**

**Figure 3:**
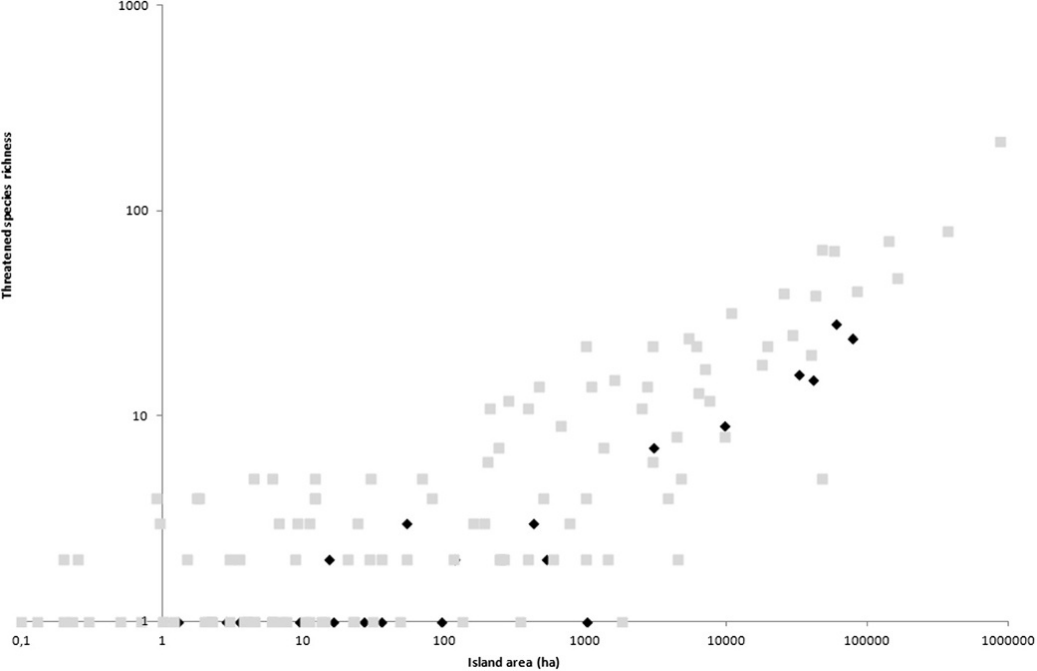
**Species-Area relationship for threatened species richness in the Aegean (grey squares) and Ionian (black diamonds) islands.**

The power model of the species-area relationship explained 76.5% and 88.8% of the variability in species richness for the Aegean and Ionian, respectively (Table 4). Greek endemic species richness and threatened species richness were strongly correlated with area in the Ionian archipelago (R^2^ = 0.888 and R^2^ = 0.919, respectively) and were significantly correlated with area for the Aegean archipelago (R^2^ = 0.558 and R^2^ = 0.591, respectively) (Table 4). Single island endemic species richness and threatened endemic species richness were only significantly correlated with area for the Aegean (not the Ionian). The lack of significance for the Ionian might be an artefact of the small number of islands with endemics in this region, and, thus, the low degrees of freedom (d.f. = 3). Given the significance of area as a driver of diversity, we compared island diversity patterns among the two archipelagos, taking island area into account. Although for total species richness, the Ionian Islands hosted a large number of species comparable to islands of similar size in the Aegean (Figure 1) for endemics species richness they hosted far fewer endemics compared to islands of similar size in the Aegean (Figure 2), with the same trend being obtained for threatened species richness (Figure 3).

**Table 4:** **Results of regressions for S, GE, RE, SIE, TS and TES with A**

Data set	Function	R ^2^	***p***
**Total species richness (S)**			
Ionian Islands (31 islands)	logS = 1.574 + 0.297 log A	0.888	0.0001
Aegean Islands (210 islands)	logS = 1.304 + 0.365 log A	0.765	0.0001
**Greek endemics (GE)**			
Ionian Islands (10 islands)	GE = 4.137 + 0.0007 A	0.888	0.0001
Aegean Islands (144 islands)	GE = 5.100 + 0.0002 A	0.558	0.0001
**Regional endemics (RE)**			
Ionian Islands (8 islands)	RE = 1.291 + 6.053E-5 A	0.526	0.05
Aegean Islands (65 islands)	RE = 3.393 + 5.606E-5 A	0.650	0.0001
**Single Island endemics (SIE)**			
Ionian Islands (4 islands)	ns		
Aegean Islands (18 islands)	SIE = -4.502 + 0.0002 A	0.923	0.0001
**Threatened species (TS)**			
Ionian Islands (20 islands)	TS = 2.097 + 0.000 A	0.919	0.0001
Aegean Islands (116 islands)	TS = 6.804 + 0.000 A	0.591	0.0001
**Threatened endemics (TES)**			
Ionian Islands (6 islands)	ns		
Aegean Islands (91 islands)	TES = 3.371 + 0.000 A	0.542	0.0001

A = Island area.

## Discussion and conclusions

This study identified both similarities and differences in the plant diversity patterns of the two Greek archipelagos, and hence the inferred mechanisms driving these patterns. While the Ionian contains fewer islands with a narrower range in elevation and size compared to the Aegean, similar biogeographic associations were observed for total plant species richness in both archipelagos. However, differences were obtained for the patterns and drivers of endemic species richness.

Island area has been found to be related to species richness in a plethora of studies [41–46]. Likewise, maximum elevation [24, 25, 30, 47, 48] and human presence [43, 49] have also been found to be associated with species richness. Our study presents one more example of island archipelagos where species richness is primarily defined by island area. In addition, we found a strong correlation between island area and maximum elevation, which further enhances this area effect indirectly. Thus, this study identified elevation as another major driver of biodiversity, supporting previous work by [25]. In addition, Panitsa *et al*. [50] hypothesized that elevation might represent both area and elements of habitat diversity, because higher elevation usually results in a more complex topography, a wider variety of micro-habitats, and a reduced influence of the sea [51]. Trigas *et al*. [52] observed that total species richness monotonically decreases with increasing elevation on large islands with high elevations (such as Crete), whereas endemic species richness has a unimodal response to elevation showing a peak at mid-elevation intervals.

When comparing the two archipelagos, we observed that, for similar sized islands, the Ionian hosts a rich array of species. Especially for small islands, it is clear that the Ionian hosts as many as, or more, species compared to islands of a similar size in the Aegean. Because islands in the Ionian have lower elevations, this phenomenon is not the confounding effect of elevation. We speculate that this phenomenon might be attributed to climate, because the Aegean Islands are subject to higher levels of summer drought, whereas the Ionian Islands have higher levels of precipitation, both annually and in summer (the driest season). However, this hypothesis requires testing in future studies.

Kallimanis *et al*. [25] showed that, in the Aegean, the biogeographic factors that influence total and endemic species richness differ. Here, we expand this observation to include the Ionian. Most striking is the fact that, although the total species richness of the Ionian Islands was equal to, or higher, than similarly sized islands in the Aegean, the Ionian Islands host considerably fewer endemic species compared to Aegean islands of comparable size.

The fairly low number of endemics in the Ionian Islands might be attributed to their land-bridge characteristics, as is also the case for the East Aegean Islands [24]. However, the recent isolation of the Ionian Islands might also be a determining factor explaining the low richness in endemic species. The Ionian Islands were isolated from the mainland during the Pleistocene, or more recently, whereas the palaeogeographic evolution of the Aegean Islands started about 23–12 million years ago [15]. Thus, there has been less time for the speciation and evolution of new species on the Ionian Islands. Most of the Ionian endemics have probably evolved before the isolation of the islands, resulting also to the low single island endemic species richness observed in the Ionian Islands. The low number of regional endemics in the small Ionian Islands could be attributed to both increased competition and increased stochastic extinction risks. In contrast, endemics are also found on islands of the Aegean that are three orders of magnitude smaller than in the Ionian. However, the isolation and size of the islands only partially explain the difference in the number of endemics between the two archipelagos, because Greek endemics are absent from small Ionian Islands compared to similar sized islands in the Aegean. Furthermore, if recent isolation is the only mechanism responsible for this difference, the absence of Greek endemics would not be expected. An alternative explanation might be the role of competition among species serving as a limiting factor. Precipitation levels are higher in the Ionian compared to the Aegean. Consequently, vegetation cover is higher and much denser in the Ionian compared to the Aegean. For instance, small islets are typically barren in the Aegean, whereas similarly sized islets in the Ionian are densely vegetated. This dense vegetation is primarily dominated by a few, common woody species that competitively exclude more ‘ecologically fragile’ and marginal endemic species. Consequently, most Ionian endemics tend to persist on rocky cliffs, which are areas of low interspecific competition. Another explanation for the difference in endemism levels between the two archipelagos might be based on the availability and diversity of suitable habitats for endemics. The habitats available to endemics is more restricted in the Ionian compared to the Aegean. The maximum elevation of islands hosting regional and single island endemic species is much lower in the Aegean Islands compared to the Ionian ones (Table 1). These explanations are by no means mutually exclusive, and might all be contributors to the different levels of endemism in the two archipelagos.

Another striking difference was the much lower number of threatened species (endemic and not endemic), present in the Ionian compared to the Aegean, even after taking island area into account. This finding highlights the importance of the Aegean for biodiversity conservation. This finding also demonstrates the lack of congruence between total plant species richness and threatened species diversity. Thus, at least in this case, these results support the hypothesis that species richness patterns are mainly driven by common species rather than threatened species [53, 54]. Compared to the Aegean, the Ionian Islands are rich in species, but host few threatened taxa. This difference might be explained by interspecific competition that is expected to limit competitive inferior species and thus make them more vulnerable. If this is the case, then, lower precipitation and associated summer drought stress in the Aegean might limit the dominance of competitively superior, common species, allowing the persistence of more vulnerable threatened species.

Despite the geomorphological similarity of the two Greek archipelagos, they have different geological histories and precipitation patterns, with the Aegean archipelago being far older and drier compared to the Ionian archipelago [14, 15, 39, 40]. Our analyses indicate that these differences strongly influence the diversity patterns observed in these two regions. Thus, when considering islands of similar size, those in the Ionian have higher species richness, but fewer endemics and threatened species, compared to the Aegean. The shorter evolutionary history of the Ionian partially explains low endemic species richness observed. However, precipitation may also have an important and complex role in driving the biodiversity patterns of these two archipelagos. The role of precipitation as a driver of biodiversity in Greece has been documented in Greek wetlands [55]. However, focus on climate change issues has led to the role of precipitation as a driver of ecological patterns being overshadowed by the role of temperature. In conclusion, this study demonstrates that while the Ionian and Aegean archipelagos fall on either side of the same landmass, the two regions exhibit different biodiversity patterns; hence, conservation effort should be tailored to each region separately.

## Methods

### Study areas - data sets

The current study was conducted on the islands and islets of the Aegean and Ionian archipelagos. The Aegean archipelago is a basin located between the Greek and Anatolian mainland. It is divided into five phytogeographical regions [9]; North Aegean (**NAe**), East Aegean (**EAe**), South Aegean (**KK**), Central Aegean (**Kik**), and West Aegean (**WAe**), (Additional file 1). We analyzed all the Aegean Islands as a single group and did not distinguish them in their phytogeographic regions due to the sample size. Some of the phytogeographical regions (e.g. NAe, WAe) would have included only a very small number of islands, a fact that makes inference difficult since many relationships appear as not significant due to the few degrees of freedom, especially for endemic diversity patterns. The Aegean archipelago contains considerably more islands and islets compared to the Ionian archipelago. The Ionian Islands form a single phytogeographical region (IoI), with all islands being directly adjacent to the mainland of western Greece. The Ionian Island of Othoni represents the westernmost point of Greece.

For the purposes of this study, we compiled two datasets of floristic information from 210 Aegean islands and islets and 31 Ionian islands and islets. The dataset for the Aegean is separated into five subsets, representing the phytogeographical regions; specifically, 95 islands and islets in the EAe, 50 in the KK, 30 in the Kik, 20 in the WAe and 15 in the NAe. For the analysis, we used published datasets, in addition to data collected by the authors (Additional file 2).

The surface area and the maximum elevation of the Aegean islands and islets included in this study ranged from 0.04 to 872900 ha and from 2 to 2456 m, respectively. For the Ionian islands and islets, the same parameters ranged from 1.3 to 78100 ha and from 12 to 1628 m, respectively. The maximum sea depth in the Aegean exceeds 2000 m (north of eastern Crete), while that of the Ionian exceeds 4000 m (west of Kefallinia and Zakinthos) [9]. The distance of the study islands and islets to the mainland in the Aegean and Ionian archipelagos ranged from 0.108 to 254 km and from 0.5 to 50.85 km, respectively.

The morphology of the coastal area is variable in both archipelagos, but generally consists of steep cliffs and a restricted coastal zone. The most extended shelves mainly exist in the northern Aegean and, to a lesser extent, in certain areas of the Ionian Sea and the eastern Aegean [56].

Both archipelagos have been sites of Greek civilization from Neolithic times to present [56]. Thus, both archipelagos have been subject to intensive human presence and activities for more than 8000 years [15]. Human presence on both archipelagos varies considerably among islands, from small islets that have never been inhabited or used for livestock grazing to islands with permanent populations that are highly impacted by tourism and grazing.

The number of endemic taxa was based on Dimopoulos *et al*. [12]. Data about the number of threatened species were based on the International Union for Conservation of Nature (IUCN) Red List of Threatened Species [57] and the Red Data Book of rare and threatened plants of Greece [11, 58]. Endemism was estimated using three hierarchical scales: (1) **Greek** endemics, including species confined to Greece, (2) **Regional** endemics (Aegean and Ionian endemics), which are defined as species that are exclusively present on the islands of one of the two studied archipelagos, and they are a subset of Greek endemics, and (3) **Single Island** endemics, which are only distributed on a single island or islet, and are also a subset of Regional endemics. In addition, the number of threatened species and the number of the threatened endemic species was considered.

The dependent variables examined for each archipelago include the numbers of (1) Total species richness (S), (2) Greek Endemic species (GE), (3) Regional Endemic species (RE), namely the Aegean Endemics (AE) and the Ionian Endemics (IE), (4) Single Island Endemic species (SIE), (5) Threatened Species (TS) and (6) Threatened Endemic Species (TES).

Eight independent variables were evaluated as potential drivers of total and endemic species richness. For both archipelagos, the values of certain geographical parameters were determined from maps of the Hellenic Military Geographical Service and Hellenic Navy; specifically, Area (ha, **A**), maximum elevation (m, **E**), shortest distance from the nearest species pool, the mainland (km, **Dm**) or inhabited island (km, **Dis**), average latitude (**Lat**) and average longitude (**Lon**). The number of geological substrata (**Geo**) was obtained from the Geological Map of Greece (at scales of 1:500000 or 1:50000). Finally, an index (6-point scale) of human impact (**HI**) was used, referring to [24] for the Aegean archipelago.

### Analysis

The relationship between biogeographic factors (A, E, Dm, Dis, Lat, Lon, Geo and HI) and plant species richness (S, GE, RE, SIE, TS and TES) in the Ionian and Aegean archipelagos was examined using Spearman rank correlation. The relationship between total and endemic species richness was also estimated. Spearman rank correlation tests were performed for each pair of independent variables for the two archipelagos to assess the degree of colinearity among the eight independent biogeographic variables.

We ran a non-parametric Spearman rank correlation test for all correlations, because one of the variables (Human Impact, HI) was ordinal and not quantitative and because the variables were not normally distributed. Islands with zero values for diversity were excluded from the analyses to avoid inflating the degrees of freedom.

Regression analyses were performed to examine the relationships of total and endemic species richness with Area (A). For all parameters, the generally used logarithmic transformation of the power function model S = cA^z^ [59] was applied to normalise the distribution of points, to satisfy the assumption of the regression analyses we employed. Spearman rank correlation tests and regression analyses were carried out with SPSS 17.0 [60].

Given the importance of area as a determinant of biodiversity, we plotted all biodiversity values (for total species richness, as well as for endemics and threatened species) against area so as to compare the two archipelagos.

## Authors’ information

Ms Eleni Iliadou is a PhD student at the Department of Environmental and Natural Resource Management of the University of Patras. Her research interests lie in the field of island plant taxonomy and diversity and on island biogeography.

Prof. Panayotis Dimopoulos is biologist and he is professor of botany and ecology in the Department of Environmental and Natural Resource Management of the University of Patras. His research interests lie in vegetation ecology and more specifically in plant biodiversity and community analysis, monitoring, assessment and mapping of natural habitat types in protected and non-protected areas.

Dr. Athanasios S. Kallimanis is assistant professor of ecology in the Department of Environmental and Natural Resource Management of the University of Patras. His research focuses on analysing the spatial pattern of biodiversity under different temporal and spatial scales with an emphasis in protected areas, vegetation dynamics and their applications.

Dr. Maria Panitsa is assistant professor of flora and phytogeography in Department of Environmental and Natural Resource Management of the University of Patras. Her research interests lie in biogeography, diversity and taxonomy of plant species of Greece focusing mainly on islands and habitat islands, as well as in the field of conservation and monitoring of plant species and natural habitats.

## Electronic supplementary material

Additional file 1: **Phytogeographical regions of Aegean and Ionian archipelagos.** (DOC 476 KB)

Additional file 2: **Literature concerning total floras of Aegean and Ionian islands and islets.** (DOC 44 KB)
