# Supplementary material for: Comparing the two Greek archipelagos plant species diversity and endemism patterns highlight the importance of isolation and precipitation as biodiversity drivers
Source: J Biol Res (Thessalon). 2014 Sep 19;21(1):16. doi: 10.1186/2241-5793-21-16 (PMC4389644; doi:10.1186/2241-5793-21-16)
Supplement: Supplementary file 1 — Additional file 1: Phytogeographical regions of Aegean and Ionian archipelagos. (DOC 476 KB) [file 40709_2014_16_MOESM1_ESM.doc]

Comparing the two Greek Archipelagos plant species diversity and endemism patterns highlight the importance of isolation and precipitation as biodiversity drivers.

# Eleni Iliadou, Athanasios S. Kallimanis, Panayotis Dimopoulos, Maria Panitsa*

Department of Environmental and Natural Resources Management, University of Ioannina, GR-30100 Agrinio, Greece.

Corresponding author: *E-mail: mpanitsa@upatras.gr, Tel: +30 26410 74187, Fax: +30 26410 74176

Current address: Department of Environmental and Natural Resources Management, University of Patras, Agrinio, Greece

**-------------------------------------------------------------------------------------------------------**


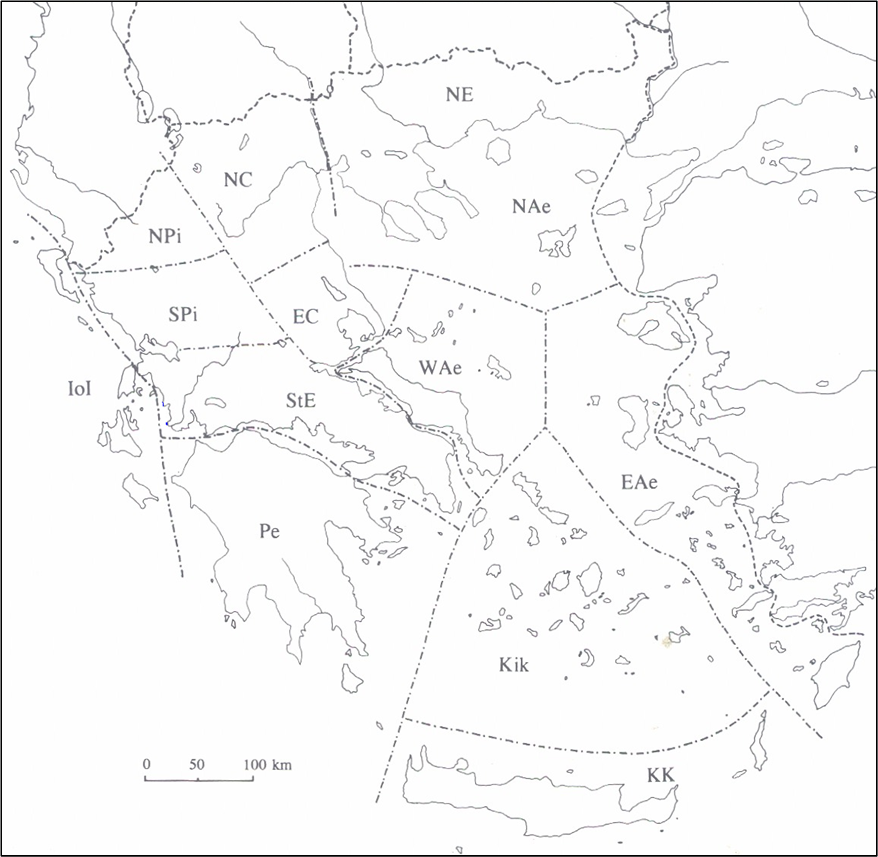


Figure 1. Division of Greece into 13 phytogeographical regions (Strid and Tan 1997). The Aegean area is divided into five phytogeographical regions: North Aegean (**NAe**), East Aegean (**EAe**), South Aegean (**KK**), Central Aegean (**Kik**), and West Aegean (**WAe**). The Ionian archipelago consists of one phytogeographical region (IoI)

**Reference**

Strid A, Tan K (ed) (1997) Flora hellenica **1.** Königstein: Koeltz.
