# Supplementary material for: Comparing the two Greek archipelagos plant species diversity and endemism patterns highlight the importance of isolation and precipitation as biodiversity drivers
Source: J Biol Res (Thessalon). 2014 Sep 19;21(1):16. doi: 10.1186/2241-5793-21-16 (PMC4389644; doi:10.1186/2241-5793-21-16)
Supplement: Supplementary file 2 — Additional file 2: Literature concerning total floras of Aegean and Ionian islands and islets. (DOC 44 KB) [file 40709_2014_16_MOESM2_ESM.doc]

Comparing the two Greek Archipelagos plant species diversity and endemism patterns highlight the importance of isolation and precipitation as biodiversity drivers.

# Eleni Iliadou, Athanasios S. Kallimanis, Panayotis Dimopoulos, Maria Panitsa*

Department of Environmental and Natural Resources Management, University of Ioannina, GR-30100 Agrinio, Greece.

Corresponding author: *E-mail: mpanitsa@upatras.gr, Tel: +30 26410 74187, Fax: +30 26410 74176

Current address: Department of Environmental and Natural Resources Management, University of Patras, Agrinio, Greece

**-------------------------------------------------------------------------------------------------------**

For the Aegean Archipelago, data concerning total floras were obtained from Bazos (2005), Bergmeier and Dimopoulos (2001, 2003), Bergmeier (2002), Bergmeier et al. (2001), Brofas et al. (2001), Burton (1991), Carlstrӧm (1987), Christodoulakis (1986, 1996, 2000), Greuter et al. (1983), Hӧner (1991), Kamari et al. (1988), Panitsa and Tzanoudakis (1998, 2001), Panitsa et al. (1994, 2003, 2004, 2006), Raus (1989, 1996a, 1996b), Snogerup and Snogerup (1987, 1993), Snogerup et al. (2001), Strid and Tan (1998), Trigas and Iatrou (2006), Tzanoudakis et al. (2006). Panitsa et al. (2006, 2010) and Kallimanis et al. (2010, 2011) present also corresponding available information concerning Aegean islands and islets' plant species richness and endemism.

For the Ionian Archipelago, the floristic data were obtained from: Baliousis and Yannitsaros (2010), Ronniger (1941), Hofmann (1968), Borkowsky (1994), Christodoulakis et al. (1988), Georgiadis (1983, 1985), Georgiadis et al. (1986), Georgiou (1988), Gutermann (1995), Hansen (1982), Phitos and Damboldt (1985), Raus (1999), Markantonatou et al. (2002), Yannitsaros et al. (1995), Strasser (2001), Iliadou, et al. (2014).

**References**

Baliousis E, Yannitsaros E (2010) Flora and vegetation of the island of Kalamos (Ionian Sea, Greece): A preliminary study. Bot Chron 20: 5–23.

Bazos I (2005) Study of the flora and vegetation of Lesvos. PhD thesis, University of Athens, Greece. (In Greek with an English summary).

Bergmeier E (2002) The vegetation of the high mountains of Crete - a revision and multivariate analysis. Phytocoenologia 32: 205–249.

Bergmeier E, Dimopoulos P (2001) Changes and limits of floristic island inventories- the Dionysades group (South Aegean, Greece) revisited. Phyton 41:277–293.

Bergmeier E, Dimopoulos P (2003) The vegetation of islets in the Aegean and the relation between the occurrence of islet specialists, island size and grazing. Phytocoenologia 33 (2-3): 447-474.

Borkowsky O (1994) Übersicht der Flora von Korfu. Braunschweiger Geobot Arb 3: 1–202.

Brofas G, Karetsos G, Panitsa M et al (2001) The flora and vegetation of Gyali island, SE Aegean, Greece. Willdenowia 31:51–70

Burton RM (1991) A check-list and evaluation of the flora of Nisiros (Dodecanese, Greece). Willdenowia 20:15–38

Carlstrӧm A (1987) A survey of the flora and phytogeography of Rodhos, Simi, Tilos and the Marmaris Peninsula (SE Greece, SW Turkey). PhD thesis, University of Lund, Sweden

Christodoulakis D (1986) Flora and vegetation of Samos. PhD thesis, University of Patras, Greece. (In Greek with an English summary)

Christodoulakis D (1996) The flora of Ikaria (Greece, E. Aegean Islands). Phyton 36:63–91

Christodoulakis D (2000) The flora of Samiopoula (E Aegean Islands, Greece): a biological, chorological and ecological analysis. Bot Chron 13:287–301

Christodoulakis D, Georgiadis T, Lösing J, Severin I (1988) Flora, Vegetation und ökologische Bedeutung der Insel Oxeiá (Ionische Inseln, Griechenland). Candollea 43:209–222.

Georgiadis T (1983) Contribution à l’ étude de la flore et de la végétation de l’ île d’ Othoni. Candollea 38:503–539.

Georgiadis T (1985) Contribution à l’étude de la flore de l’île Erikoussa (Grèce). [Contribution to the study of the flora of Erikoussa (Greece).]. Willdenowia 15:203–210.

Georgiadis T, Iatrou G, Georgiou O (1986) Contribution à l’étude de la flore et de la végétation de l’île de Paxi, Grèce. [Contribution to the study of flora and vegetation of the island of Paxos, Greece.]. Willdenowia 15:567–602.

Georgiou O (1988) The flora of Kerkira (Ionian Islands, Greece). Willdenowia 17:87–101.

Greuter W, Pleger R, Raus Th (1983) The vascular flora of the Karpathos island group (Dodecanesos, Greece). A preliminary checklist. Willdenowia 13:43–78

Gutermann W (1995) Catalogus Abbreviatus Plantarum Vascularium Ex Ionii Insulis Cognitarum. Instituto Botanico Universitatis Vindobonensis. Vindobonae.

Hansen A (1982) Additions to and Notes on the Flora of Corfu and Vidos (Ionian Islands, Greece). Bot Chron 2:18–49.

Hofmann U (1968) Untersuchungen an Flora und Vegetation der Ionischen Insel Levkas. Vierteljahrsschrift der Naturforschenden Gesellschaft in Zuerich 113:209–256.

Höner D (1991) Mehrjährige Beobachtungen kleiner Vegetationsflächen im Raume von Karpathos (Nomos Dhodhekanisou, Griechenland). Diss Bot 173:1-185.

Iliadou E, Panitsa M, Raus T, Dimopoulos P (2014) Flora and factors affecting species diversity in protected "Natura 2000" sites of the Ionian area: The Echinades islet group (Greece). Willdenowia 44: 121 – 136.

Kallimanis AS, Bergmeier E, Panitsa M, Georghiou K, Delipetrou P, Dimopoulos P (2010) Biogeographic determinants for total and endemic species richness in a continental archipelago. Biodivers Conserv 19:1225-1235.

Kallimanis AS, Panitsa M, Bergmeier E, Dimopoulos P (2011) Examining the relationship between total species richness and single island palaeo- and neo-endemics. Acta Oecol Int J Ecol 37:65-70.

Kamari G, Phitos D, Snogerup B et al (1988) Flora and vegetation of Yioura, N Sporades, Greece. Willdenowia 17:59–85.

Markantonatou A, Sarlis G, Constantinidis Th (2002) The flora of Ithaki island (Ionia Sea, Greece): a work in progress. Proceedings of the 9^th^ conference of the Hellenic Botanical Society, Cephalonia, Greece, 9-12 May 2002 (in Greek).

Panitsa M, Bazos I, Dimopoulos P et al (2004) Contribution to the study of the flora and vegetation of the Kithira island group: offshore islets of Kithira (S Aegean, Greece). Willdenowia 34:101–115.

Panitsa M, Dimopoulos P, Iatrou G, Tzanoudakis D (1994) Contribution to the study of the Greek flora: flora and vegetation of the Enousses (Oinousses) islands (E Aegean area). Flora 189:367–374.

Panitsa M, Snogerup B, Snogerup S, Tzanoudakis D (2003) Floristic investigation of Lemnos island (NE Aegean area, Greece). Willdenowia 33:79–105

Panitsa M, Trigas P, Iatrou G, Sfenthourakis S (2010) Factors affecting plant species richness and endemism on land-bridge islands – An example from the East Aegean archipelago. Acta Oecol Int J Ecol Acta 36:431-437.

Panitsa M, Tzanoudakis D (1998) Contribution to the study of the Greek flora: flora and vegetation of the E Aegean islands Agathonisi and Pharmakonisi. Willdenowia 28:95–116.

Panitsa M, Tzanoudakis D (2001) A floristic investigation of the islet groups Arki and Lipsi (East Aegean area, Greece). Folia Geobot 36:265–279.

Panitsa M, Tzanoudakis D, Triantis K.A, Sfenthourakis S (2006) Patterns of species richness on very small islands: the plants of the Aegean archipelago. J Biogeogr 33:1223-1234.

Phitos D, Damboldt J (1985) Die Flora der Insel Kefallinia (Griechenland). Bot Chron 5:1–204.

Raus T (1989) Die Flora von Armathia und der Kleininseln um Kasos (Dodekanes, Griechenland). Bot Chron 9:19–39.

Raus T (1996a) Additions and amendments to the flora of the Karpathos island group (Dodekanesos, Greece). Bot Chron 12:21–53.

Raus T (1996b) Flora von Paros und Antiparos (Kykladen, Griechenland). Ann Naturhist Mus Wien 98(B Suppl):237–278.

Raus T (1999) Heinrich Kuhbier und die geobotanische Erforschung der Insel Korfu (Jonische Inseln, Griechenland). Abh Naturwiss Verein Bremen 44:397–416.

Ronniger K (1941) Flora der Insel Zante. Verh Zool- Bot Ges Wien 88/89:13–108.

Snogerup S, Snogerup B (1987) Repeated floristical observations on islets in the Aegean. Plant Syst Evol 155:143-164.

Snogerup S, Snogerup B (1993) Additions to the flora of Samos, Greece. Flora Mediterr 3:211-222.

Snogerup S, Snogerup B, Phitos D, Kamari G (2001) The flora of Chios island (Greece). Bot Chron 14:5-199.

Strasser W (2001) Zur Flora der griechischen Indel Lefkas (Lefkada) als Ergänzungen: Aufnahmen auf dem südlichen Peloponnes und in NW-Griechenland. - Steffisburg: W.Strasser, 2001.

Strid A, Tan K (eds) (1998) Flora and vegetation of North East Greece, including the islands of Thasos and Samothraki. Report of a student excursion from the University of Copenhagen May 17–31, 1997. Botanical Institute, Copenhagen

Trigas P, Iatrou G (2006) The local endemic flora of Evvia (W Aegean, Greece). Willdenowia 36:257–270.

Tzanoudakis D, Panitsa M, Trigas P, Iatrou G. (2006) Floristic and phytosociological investigation of the Aegean islands and islets: Antikythera islets’group (SW Aegean area, Greece). Willdenowia 36:285–301.

Yannitsaros A, Vallianatou I, Bazos I, Constantinidis T (1995) Flora and vegetation of Strofades islands (Ionian Sea, Greece). Hellenic Society for the Protection of Nature, Athens:3-26.
